# Vitamin and trace element concentrations in infants and children with chronic kidney disease

**DOI:** 10.1007/s00467-020-04536-0

**Published:** 2020-04-14

**Authors:** Triona Joyce, Pernille Rasmussen, Nabil Melhem, Joanna Clothier, Caroline Booth, Manish D Sinha

**Affiliations:** 1 Department of Nutrition and Dietetics, Evelina London Children’s Hospital, Guys & St Thomas’ NHS Foundation Trust, Westminster Bridge Road, London, SE1 7EH UK; 2 Department of Paediatric Nephrology, Evelina London Children’s Hospital, Guys & St Thomas’ NHS Foundation Trust, 3rd Floor Beckett House, Westminster Bridge Road, London, SE1 7EH UK; 3 Kings College London, London, UK

**Keywords:** Chronic kidney disease, Vitamins, Trace elements, Blood monitoring

## Abstract

**Background:**

There are limited data regarding vitamin and trace element blood concentrations and supplementation needs in children with non-dialysis stages 3–5 of chronic kidney disease (CKD).

**Methods:**

Retrospective cross-sectional review for nutritional blood concentrations measured over a recent 2-year period. In our CKD clinics, nutritional bloods including copper, zinc, selenium and vitamin A, vitamin E, active vitamin B_12_ and folate are monitored annually. Vitamin D status is monitored every 6–12 months.

**Results:**

We reviewed 112 children (70 boys) with median (IQ1, IQ3) age 8.97 (4.24, 13.80) years. Estimated median (IQ1, IQ3) GFR (mL/min/1.73 m^2^) was 28 (21, 37). Vitamin A, active vitamin B_12_ and vitamin E concentrations were within normal range in 19%, 23% and 67% respectively, with all others being above normal range. Vitamin D blood concentrations were within desired range for 85% (15% had low levels) and folate blood concentrations were within normal range in 92%, with the remainder above or below target. For trace elements, 60%, 85% and 87% achieved normal ranges for zinc, selenium and copper respectively. Deficiencies were seen for zinc (35%), copper (7%), folate (3%) and selenium (1%), whilst 5%, 6% and 14% had zinc, copper and selenium levels above normal ranges.

**Conclusions:**

Several vitamin and trace element blood concentrations were outside normal reference ranges. Monitoring vitamin D and zinc blood concentrations is indicated due to the percentages with low levels in this group. Targeted vitamin and trace element supplementation should be considered where indicated rather than commencing multivitamin and/or mineral supplementation.

**Graphical abstract:**
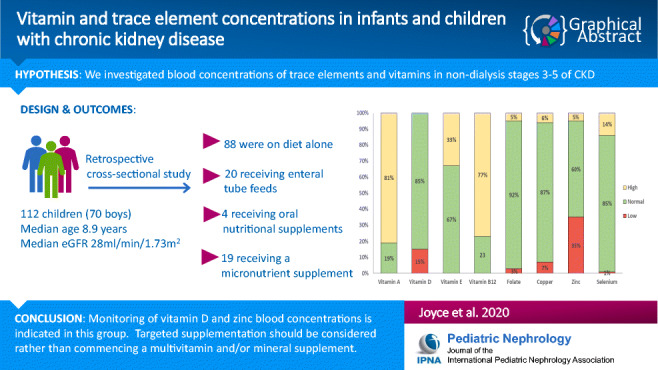
Vitamin and trace element concentrations in infants and children with non-dialysis chronic kidney disease

**Electronic supplementary material:**

The online version of this article (10.1007/s00467-020-04536-0) contains supplementary material, which is available to authorized users.

## Introduction

Children with chronic kidney disease (CKD) may be at risk of micronutrient deficiencies due to multiple factors, which may include inadequate intake as a result of dietary restrictions, anorexia or interference with absorption, metabolism and excretion [1]. The provision of adequate quantities of micronutrients is important due to their role in paediatric growth and development [1].

K/DOQI guidelines have recommended that the provision of the dietary reference intake (DRI) for several micronutrients, including copper, zinc and vitamins A, E and C and folic acid should be considered in children with CKD stages 2 to 5 [1]. In the UK, recommended nutrient intakes (RNI) are estimates of requirements for the healthy population and may not meet the needs of children with CKD [2]. With regard to vitamin and trace element supplementation, K/DOQI guidelines suggest supplementation if dietary intake does not meet 100% of the DRI or where there is clinical evidence of a deficiency [1]. However, it has been acknowledged that micronutrient deficiencies progress slowly in most cases with clinical manifestations indistinguishable from those of CKD [3]. With the exception of vitamin D (25 hydroxy vitamin D), there are limited data regarding micronutrient and trace element status, requirements and supplementation needs in infants and children with CKD.

In the CKD clinics at the Evelina London Children’s Hospital, nutritional bloods including copper, zinc, selenium and vitamin A, vitamin E, active vitamin B_12_ (holo-transcobalamin) and folate are monitored annually and vitamin D (25 hydroxy vitamin D) status every 6–12 months. Infants and children are not routinely supplemented with micronutrient supplements. Our objective in this study was to evaluate the blood concentrations of trace elements and vitamins in children with non-dialysis stages 3–5 of CKD, including those on nutritional supplementation.

## Methods

We performed a retrospective cross-sectional review of nutritional blood concentrations in children attending the non-dialysis CKD clinics at our centre over a recent 2-year period. Additional data collected included patient demographics (age, gender, ethnicity), primary renal diagnosis and micronutrient supplementation. We categorized patients into sub-groups by their method of diet intake as (i) diet alone: those who were taking diet without any nutritional supplementation; (ii) enteral tube feed: those receiving some or all of their nutrition via nasogastric or gastrostomy tubes; and (iii) oral nutritional supplements (ONS) (also known as sip feeds): those taking nutritional supplements orally. Additionally, children on any micronutrient supplementation (vitamin and/or trace elements) were identified. The micronutrient supplements included ABIDEC® multivitamin drops, Dalivit® drops, FruitiVits®, Floradix® liquid iron and vitamin formula, Forceval® capsules, Millhouse Jelly Bear® multivitamins, Wellteen® Her/Him, Wellkid® multivitamin liquid and Wellbaby® multivitamin liquid (please see online Supplementary Table for specific content).

All measurements were obtained using electronic patient records. Information on the vitamin and trace element content of the enteral tube feeds was obtained using the software program Electronic Dietetic Manager (EDM 2000™, MicroMan2000 Ltd, PO box 3721, Newport Pagnell, UK).

Data are displayed as number (percentage), median and interquartile range (IQR) where appropriate for all children. Values were reported as ‘above’, ‘within range’ or ‘normal’, and ‘below’ for all reported micronutrients. Micronutrient intakes were compared with the RNI. The Kruskal-Wallis test was used to compare median ages and micronutrient concentrations between the different CKD stages. Chi-square test and ANOVA were used to compare groups. The Mann-Whitney test was used to compare non-parametric data. Analysis was performed using SPSS version 25 (SPSS Inc., Chicago, IL) and *p* < 0.05 was taken as significant.

## Results

A total of 112 children were included during the study period with a median (IQ1, IQ3) age of 8.97 (4.24, 13.80) years. Estimated median (IQ1, IQ3) GFR (mL/min/1.73 m^2^) was 28 (21, 37). Patient characteristics for the total group and by CKD stages 3 to 5 are shown in Table 1. Eighty-eight of 112 (78.6%) children were on diet alone, 20 (17.8%) on enteral tube feeds and four (3.6%) on ONS. Overall, 19 of 112 (17%) children were receiving a micronutrient supplement, including 17 of 88 (19.3%) who were on diet alone and 2 of the 20 (10%) children receiving enteral tube feeds. There were no patients on micronutrient supplements in the ONS group.

**Table 1:** Patient characteristics for the total study group and by chronic kidney disease stages 3–5

	Total (*n* = 112)	Stage 3 (*n* = 50)	Stage 4 (*n* = 48)	Stage 5 (*n* = 14)	
Age in years, median (IQ1, IQ3)	8.97 (4.24, 13.80)	9.08 (4.62, 12.90)	9.35 (4.42, 14.66)	6.12 (0.94, 9.97)	*p* = 0.139
Gender, *n* (%)					
Girls	42 (37%)	18 (36%)	20 (42%)	4 (29%)	*p* = 0.644
Boys	70 (63%)	32 (64%)	28 (58%)	10 (71%)	
Ethnicity, *n* (%)					
White	68 (61%)	34 (68%)	25 (52%)	9 (64.3%)	*p* = 0.33
Black	14 (12%)	4 (8%)	9 (19%)	1 (7.1%)	
Asian	14 (12%)	4 (8%)	8 (17%)	2 (14.3%)	
Mixed	13 (12%)	5 (10%)	6 (12%)	2 (14.3%)	
Other	3 (3%)	3 (6%)	-	-	
Causes of CKD, *n* (%)					*p* = 0.327
Glomerular disease	3 (3%)	2 (4%)	1 (2%)	-	
Tubulo-interstitial disease	5 (4%)	-	4 (8%)	1 (7.1%)	
Metabolic disease	7 (6%)	6 (12%)	1 (2%)	-	
Renovascular disease	12 (11%)	5 (10%)	7 (15%)	-	
Obstructive uropathy	27 (24%)	11 (22%)	13 (27%)	3 (21.4%)	
Renal dysplasia ± reflux nephropathy	55 (49%)	25 (50%)	20 (42%)	10 (71.4%)	
Polycystic	1 (1%)	-	1 (2%)	-	
Uncertain	2 (2%)	1 (2%)	1 (2%)	-	
Estimated GFR (mL/min/1.73 m^2^), median (IQ1, IQ3)	28 (21, 37)	38 (35, 44)	24 (19, 27)	12 (11, 13)	*p* < 0.01
Micronutrient supplement, *n* (%)	19 (17%)	9 (18%)	7 (15%)	3 (21%)	*p* = 0.807
Enteral feed, *n* (%)	20 (18%)	5 (10%)	13 (27%)	2 (14%)	*p* = 0.082

*IQ1* interquartile 1, *IQ3* interquartile 3, *CKD* chronic kidney disease

**Fig. 1:**
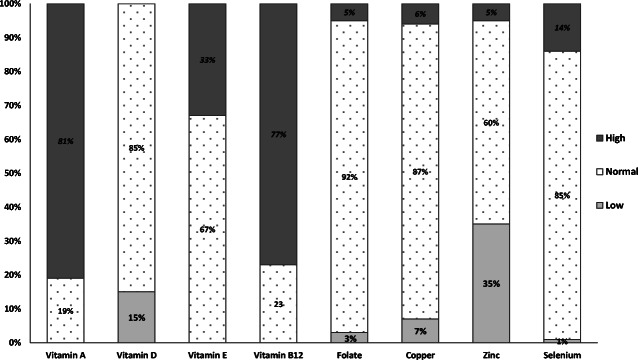
Percentage of mean vitamin and trace element concentrations that were below, within and above their reference ranges

Ninety-eight children had nutritional blood results for all eight vitamins and trace elements. Of these, the majority (*n* = 93) had ≥ 1 vitamin or trace element abnormality outside of the normal reference range and 52 (53%) had ≥ 1 of both vitamin and trace element abnormalities. Table 2 shows the median (IQI, IQ3) blood concentrations of vitamin and trace elements in the total group and by CKD stages 3–5. The percentages of vitamin and trace element concentrations below, within and above their reference ranges are shown in Fig. 1. The majority of children had adequate vitamin E concentrations, with significantly increasing concentrations with worsening stages of CKD (*p* = 0.019). Table 3 shows patient characteristics and vitamin and trace element concentrations in those on diet alone versus those receiving nutritional supplementation (patients in the enteral tube feed and ONS sub-groups). The laboratory reference ranges for vitamin and trace elements at our unit are shown in Tables 4 and 5.

**Table 2:** Median (IQ1, IQ3) blood concentrations of vitamins and trace elements in the total group and by chronic kidney disease stages 3–5

	Total (*n* = 112)		Stage 3 (*n* = 50)		Stage 4 (*n* = 48)		Stage 5 (*n* = 14)		
	*n*	Median (IQ1, IQ3)	*n*	Median (IQ1, IQ3)	*n*	Median (IQ1, IQ3)	*n*	Median (IQ1, IQ3)	
Vitamins									
Vitamin A (μmol/L)	101	2.52 (2.06, 3.24)	43	2.45 (1.97, 2.78)	44	2.57 (2.25, 3.41)	14	2.70 (2.17, 3.94)	*p* = 0.122
Vitamin D (nmol/L)	112	73 (57, 89)	50	70 (55, 92)	48	75 (55, 88)	14	77 (61, 106)	*p* = 0.652
Vitamin E (μmol/L)	101	29.8 (25.3, 34.7)	43	27.4 (25.2, 30.4)	44	31.9 (26.5, 37.1)	14	33.3 (28.5, 39.1)	p = 0.019
Trace elements									
Copper (μmol/L)	104	17 (13, 20)	45	16 (14, 19)	45	17 (13, 21)	14	17 (15, 20)	*p* = 0.867
Zinc (μmol/L)	103	11.6 (10.4, 12.8)	44	11.4 (10.4, 12.8)	45	11.9 (9.8, 13.3)	14	12.2 (11.0, 12.8)	*p* = 0.917
Selenium (μmol/L)	104	1.17 (1.02, 1.33)	45	1.17 (1.04, 1.28)	45	1.14 (1.01, 1.33)	14	1.21(0.95, 1.43)	*p* = 0.769

*IQ1* interquartile 1, *IQ3* interquartile 3

As vitamin B_12_ and folate results above range were reported as > 128 pmol/L and > 20μg/L respectively values for these are not displayed

**Table 3:** Patient characteristics and vitamin and trace element concentrations in the diet alone and enteral tube feed and oral nutritional supplement (ONS) groups

	Diet group (*n* = 88)	Enteral tube feed and ONS groups (*n* = 24)	
Age in years, median (IQ1, IQ3)	9.44 (4.20, 14.04)	7.60 (4.24, 11.73)	*p* = 0.194
Gender, *n* (%)			
Girls	30 (34%)	12 (50%)	*p* = 0.154
Boys	58 (66%)	12 (50%)	
Ethnicity, *n* (%)			
White	55 (63%)	13 (54.1%)	*p* = 0.712
Black	11 (13%)	3 (12.5%)	
Asian	10 (11%)	4 (16.7%)	
Mixed	9 (10%)	4 (16.7%)	
Other	3 (3%)	-	
Causes of CKD, *n* (%)			*p* = 0.609
Glomerular disease	3 (3%)	-	
Tubulo-interstitial disease	4 (5%)	1 (4%)	
Metabolic disease	5 (6%)	2 (8%)	
Renovascular disease	9 (10%)	3 (13%)	
Obstructive uropathy	21 (24%)	6 (25%)	
Renal dysplasia ± reflux nephropathy	44 (50%)	11 (46%)	
Polycystic	-	1 (4%)	
Uncertain	2 (2%)	-	
Estimated GFR (mL/min/1.73 m^2^), median (IQ1, IQ3)	29 (22, 38)	25 (17, 31)	*p* = 0.119
Vitamins			
Vitamin A (μmol/L), median (IQ1, IQ3) (*n*)	2.52 (2.04, 3.24) (81)	2.53 (2.28, 3.22) (20)	*p* = 0.779
Vitamin D (nmol/L), median (IQ1, IQ3) (*n*)	66 (54, 87) (88)	84 (77, 102) (24)	*p* = 0.001
Vitamin E (μmol/L), median (IQ1, IQ3) (*n*)	28.9 (25.1, 33.9) (81)	34.1 (28.1, 39.5) (20)	*p* = 0.008
Trace elements			
Copper (μmol/L), median (IQ1, IQ3) (*n*)	16 (13, 19) (83)	19 (16, 23) (21)	*p* = 0.017
Zinc (μmol/L), median (IQ1, IQ3) (*n*)	11.5 (10.4, 12.6) (82)	12.8 (10.25, 14.55) (21)	*p* = 0.064
Selenium (μmol/L), median (IQ1, IQ3) (*n*)	1.14 (1.02, 1.29) (83)	1.26 (1.00, 1.52) (21)	*p* = 0.128

*IQ1* interquartile 1, *IQ3* interquartile 3

**Table 4:** Laboratory reference ranges for folate, vitamin D, vitamin B_12_, copper, zinc and selenium

Variable	Normal range
Folate	3.1–20.5 μg/L
Vitamin D	< 30 nmol/L (deficiency), 30–50 nmol/L (insufficiency)
Vitamin B_12_	25–108 pmol/L
Selenium	0.44–1.43 μmol/L
Zinc	11–19 μmol/L
Copper	1.4–7.2 μmol/L (0–4 months)
	3.9–17.3 μmol/L (4–6 months)
	7.9–20.5 μmol/L (7–12 months)
	12–25 μmol/L (> 1 year)

**Table 5:** Laboratory reference ranges for vitamin A and vitamin E

Age (years)	Vitamin A (μmol/L)	Vitamin E (μmol/L)
0–2	0.49–1.43	0.0–25.0
3–5	0.56–1.47	7.0–30.1
6–8	0.66–2.00	10.0–34.8
9–11	0.77–2.06	13.9–32.5
12–13	0.84–2.20	10.9–34.8
14–15	0.94–2.65	13.9–32.5
16+	1.40–3.84	11.6–41.8

### Diet alone group

Patient characteristics of the 88 children in this group are shown in Table 3. Vitamin D and vitamin E concentrations were lower in those on diet alone when compared with those receiving nutritional supplementation (vitamin D [66 (54, 87) versus 84 (77, 102) nmol/L, *p* = 0.001] and vitamin E [28.9 (25.1, 33.9) versus 34.1 (28.1, 39.5) μmol/L, *p* = 0.008], respectively).

#### Vitamins

Active vitamin B_12_, vitamin A and vitamin E blood concentrations were within range in 29% (*n* = 24/84), 21% (*n* = 17/81) and 74% (*n* = 60/81) respectively, with all others above the normal range. Ten of the children with raised active vitamin B_12_ blood concentrations were receiving micronutrient supplements which contained vitamin B_12_ providing between 100 and 1000% respectively of the reference nutrient intake (RNI) for vitamin B_12_. Four children had high folate blood concentrations and two of these were receiving micronutrient supplements which contained folate providing 67% (also receiving a folic acid supplement) and 160% of the RNI for folate. One other child was receiving a folic acid supplement. Thirteen children with high vitamin A blood concentrations were receiving micronutrient supplements which contained vitamin A providing 33–375% of the RNI (≤ 50% of the RNI in 6 children). Vitamin D blood concentrations were deficient in four and insufficient in 14 patients. Seven patients received cholecalciferol at some point during the study. One further patient received a vitamin D–containing supplement but no cholecalciferol.

#### Trace elements

Copper blood concentrations were below the reference range in five children, three of which were only marginally below the reference range. Two of the children with low copper blood concentrations (5 and 8 μmol/L) were receiving a micronutrient supplement containing copper providing 21% and 100% of the RNI for copper respectively. Five children had blood copper concentrations above the normal reference range, one of whom was receiving supplement providing 25% of the RNI for copper. Zinc blood concentrations were below the normal reference range in 34% (*n* = 28/82) of children. Six children with zinc concentrations below the normal range and one with high zinc blood concentrations (45.5 μmol/L) were receiving micronutrient supplements containing zinc providing 50–158% (50–60% of the RNI in 3 patients) and 62% of the RNI for zinc respectively. Five other children were receiving zinc supplements to treat low zinc blood concentrations. Selenium blood concentrations were above the normal range in eight (10%) patients, of whom one child was receiving a micronutrient supplement which contained selenium providing 137% of the RNI. Only one child had a low selenium blood concentration (0.32 μmol/L) with the supplement providing 92% of the RNI.

### Enteral tube feed group

Twenty children were receiving enteral tube feeds, including 11 boys, median age 7.32 (4.24, 9.61) years, of whom 19 had minimal or no food intake. None of the children was receiving an enteral tube feed that was entirely a paediatric renal-specific feed. Active vitamin B_12_, vitamin A, vitamin E, selenium and folate blood concentrations were within range in 5%, 6%, 41%, 72% and 95% respectively, with all others above the normal range.

#### Vitamins

All of the children who were receiving enteral tube feeds had high active vitamin B_12_ blood concentrations. The enteral tube feed for only one of these was providing less than the RNI for vitamin B_12_ (90%). In all others, the enteral tube feeds provided between 100% and almost 5 times the RNI for vitamin B_12_. Two other children were receiving a multivitamin that contained vitamin B_12_ which increased their vitamin B_12_ intake from approximately 190% and 370% of the RNI to greater than 4 and 5 times the RNI respectively. The majority of children had raised vitamin A blood concentrations (*n* = 16/17) with feeds providing 45 to 205% of the RNI (< 100% of the RNI in 8 children). Two children were receiving a micronutrient supplement that contained vitamin A which provided 40% and 33% of the RNI for vitamin A, increasing their total vitamin intake to 114% and 238% respectively. All of the children receiving enteral feeds had normal vitamin D blood concentrations.

#### Trace elements

Zinc blood concentrations were below the normal range in 44% (*n* = 8/18) of children, one of whom (zinc concentration 5.5 μmol/L) was also receiving a micronutrient supplement which contained zinc. The enteral feed for the latter was providing 9 mg zinc daily (RNI 7 mg/day). Zinc blood concentrations were marginally below the normal range (10 to 10.8 μmol/L) in three patients with enteral feeds providing 46% (supplementary feed providing ~ 14% of their calorie needs), 100% and 107% of the RNI for zinc. The enteral feeds for the remaining patients provided between 71 and 167% of the RNI for zinc. One child had a marginally low copper blood concentration (11 μmol/L) with enteral feeds providing 135% of the RNI for copper.

### ONS group

In the four children in this group, zinc, folate, active vitamin B_12_ and vitamin D blood concentrations were all within range. Vitamin A, vitamin E and selenium blood concentrations were within the normal range for 33% of patients with the remainder above the normal range. No child was receiving cholecalciferol. Only one child had a marginally low copper blood concentration (11 μmol/L) with their ONS providing 73% of the RNI for copper.

## Discussion

We report the largest study of vitamin and trace element concentrations in non-dialysis children with residual renal function. We observed vitamin A (81%) and active vitamin B_12_ (77%) blood concentrations were above the normal reference ranges in the majority whilst vitamin D and zinc blood concentrations were below the normal reference ranges in 15% and 35% respectively. Blood concentrations of vitamin E (67%), folate (92%), copper (87%) and selenium (85%) were within the normal ranges for the majority of patients with only a small percentage of patients with low selenium (1%) and copper (7%) blood concentrations. Furthermore, except for vitamin E, there were no significant differences in the mean blood concentrations of vitamin and trace elements across stages 3–5 of CKD. No symptoms of vitamin or trace element excesses/deficiencies were formally recorded in this study. There was no evidence of symptoms relating to low or high blood concentrations of vitamins or trace elements.

Vitamin A blood concentrations are known to be raised in those with impaired renal function and it has been shown that hypervitaminosis A is seen early in children with CKD [4]. High blood concentrations of vitamin A can lead to hypercalcaemia [2, 4], hyperlipidaemia and anaemia. Manickavasagar et al. [4] observed a 13% rise in serum retinol blood concentrations for every 10 mL/min/1.73 m^2^ decline in renal function. They also noted increased dietary vitamin A intake, in particular from supplementary feeds, was associated with hypervitaminosis. Whilst Rees and Shaw [2] noted that common UK practice is not to exceed 200% of the RNI from the diet and/or supplements, Manickavasagar et al. [4] based on their data, suggested that vitamin A intakes below the current RNI may be safe. In our study, of the children receiving feeds (and no multivitamins) (*n* = 15), feeds provided less than 100% of the RNI for vitamin A in 53%; however, only one of these children had a normal vitamin A blood concentration with all others above the normal reference range.

Most children achieved normal vitamin E blood concentrations with the remaining blood concentrations above the normal reference range. Vitamin E concentrations significantly increased with worsening stage of CKD suggesting vitamin E blood concentrations increase as kidney function decreases. Furthermore, we previously showed a higher percentage of vitamin E blood concentrations above the normal reference range (87%) in children on maintenance dialysis [5]. In the current study, the majority of children (92%) had folate blood concentrations within the normal reference range with only a small number receiving a micronutrient supplement that contained folate (*n* = 10).

Active vitamin B_12_ concentrations were above the normal ranges in 77% of children regardless of whether they were receiving a multivitamin supplement or enteral tube feed containing vitamin B_12_. The majority of the patients received a non-renal-specific paediatric feed/formula to help meet their nutritional requirements which provided, in most cases, excessive amounts of vitamin B_12_ compared with the RNI. Whilst modification of their enteral feed recipe to reduce the vitamin B_12_ content without impacting upon the feed overall nutritional quality may prove difficult, avoidance of additional sources of vitamin B_12_, such as multivitamin supplements, is important. The K/DOQI report recommends the provision of dietary vitamin B_12_ intakes of at least 100% of the DRI with supplementation if vitamin B_12_ intakes do not meet this [1]. In our study, one of the children who was feed dependent received less than the RNI for vitamin B_12_ (90%). Despite this, they had a high active vitamin B_12_ blood concentration. As a result, where there are concerns over vitamin B_12_ intake/status, it may be worthwhile testing vitamin B_12_ blood concentrations before supplementing with vitamin B_12_.

It is well recognized that vitamin D deficiency is common in children with CKD and contributes to CKD mineral and bone disorder and guidance on the vitamin D monitoring and supplementation in this population has recently been published [6]. In our study, vitamin D blood concentrations were within the desired range in 85% of children and cholecalciferol was appropriately commenced where indicated. Eighteen children were receiving a micronutrient supplement that contained vitamin D, of whom two had insufficient vitamin D blood concentrations (33 mmol/L and 49 mmol/L) without which it is likely that their vitamin D concentrations would have been lower.

It has been reported that in CKD, derangements in zinc homeostasis may occur due to several factors including disturbed renal excretion and altered protein metabolism [7]. Symptoms of zinc deficiency include impaired smell and taste [7], anorexia and impaired wound healing [1, 7]. Furthermore, zinc deficiency can negatively impact upon growth [1, 7]. As highlighted by Yonova et al. [7], some of the symptoms of CKD resemble those of zinc deficiency.

The K/DOQI [8] has previously recommended monitoring zinc and copper intakes every 4 to 6 months and supplementing where intakes are low or in those where there is laboratory or clinical evidence of deficiency. Only seven children had low copper blood concentrations with the majority (71%) of these blood concentrations only marginally below the reference range. Whilst it has been reported that a deficiency of copper can result in neutropenia and osteoporosis and can have a negative impact upon growth, an excess of zinc may aggravate marginal copper deficiency [1].

Although it is acknowledged that zinc blood concentrations can be low in CKD [7], few studies have investigated zinc blood concentrations in children with CKD, particularly in those with non-dialysis stages of CKD. The results of our study showed that zinc blood concentrations were below the normal reference range in 36 children, six of whom were receiving a micronutrient supplement which contained zinc. The latter provided approximately 50–60% of the RNI for zinc without which their zinc levels would likely have been even lower. There was no consistent evidence that providing the RNI for zinc in children receiving enteral tube feeds results in normal zinc blood concentrations. For example, eight children with low blood zinc concentrations were receiving enteral tube feeds (one also received a micronutrient supplement which contained zinc) which in 50% of these provided ≥ 100% of the RNI for zinc. In comparison, the enteral tube feeds of 43% of children who had normal zinc blood concentrations provided only 62–88% of the RNI for zinc.

With regard to selenium blood concentrations, the majority had normal concentrations (85%) with only one child exhibiting low selenium blood concentrations indicating that routine monitoring of this trace element is not required in children with CKD who are conservatively managed. These findings are similar to those reported previously by us, where the majority of children on maintenance dialysis had normal selenium blood concentrations (89%) [5], indicating that monitoring or supplementation of selenium is not required in children with CKD pre or whilst on maintenance dialysis.

The studies by Esmaeili and Rakhshanizadeh [9] and Esfahani et al. [10] reported no significant difference between the serum zinc and copper concentrations between healthy children and those with CKD receiving conservative management. Esmaeili and Rakhshanizadeh [9] also noted no significant difference between serum concentrations of selenium between healthy children and those with CKD receiving conservative management. Youssef et al. [11] reported that zinc and copper concentrations were significantly lower in children with CKD (stages 3 and 4) on conservative management, compared with healthy children. However, none of these studies provided information on the percentage of these trace elements within or outside the normal reference ranges. In addition, the number of children with conservatively management CKD in these studies was small and no information was provided on whether children were in receipt of oral nutritional supplements or enteral tube feeds.

There are several limitations to our study findings. These include the following: (1) Whilst we have discussed the RNI for several micronutrients in this study, it may be misleading to compare children with non-dialysis stages of CKD against these. We highlight here the relative lack of data and guidance on the micronutrient needs specific to this paediatric group; (2) Due to the retrospective nature of this study, data was not available for dietary intake at the time of the measurements. However, there are numerous known limitations with dietary intake assessments including errors in recall and misreporting of food portion sizes which can impact upon interpretation of micronutrient intakes; (3) As several micronutrients have age-specific ranges (such as copper and vitamins A and E), interpretation of results adds further difficulty. It is important to state that vitamin B_12_ levels do not accurately reflect intake and if there any clinical concerns of B_12_ deficiency, levels should be measured. In this study, we have measured ‘active vitamin B_12_ (holo-transcobalamin)’ concentrations as this is a more reliable marker of low vitamin B_12_ status than that indicated by low level of serum vitamin B_12_; (4) Data on adherence to prescribed supplements and over the counter micronutrients was not available; (5) Interpretation of data from our single-centre UK-based study needs to be factored against local childhood CKD population differences, their dietary intake, micronutrient supplements/supplementation practices and differences in enteral tube feeds/feed recipes.

## Conclusions

The results of this largest study to date showed that the majority of children had vitamin A and active vitamin B_12_ blood concentrations above the normal reference ranges. Vitamin E alone showed significant difference in concentrations with worsening stage of CKD. We recommend targeted vitamin and trace element supplementation only. Monitoring of vitamin D (6 to 12 monthly) and zinc (3 to 6 monthly) blood concentrations is indicated due to higher proportions of patients with low levels in this group. Further research is required to expand this knowledge base including guidance on systematic monitoring and supplementation needs of vitamin and trace elements in infants and children with CKD.

## Electronic supplementary material

ESM 1 (PPTX 109 kb).

ESM 2 (DOCX 17 kb).
