# Supplementary material for: Vitamin and trace element concentrations in infants and children with chronic kidney disease
Source: Pediatr Nephrol. 2020 Apr 14;35(8):1463–70. doi: 10.1007/s00467-020-04536-0 (PMC7316696; doi:10.1007/s00467-020-04536-0)
Supplement: Supplementary file 1 — (PPTX 109 kb). [file 467_2020_4536_MOESM1_ESM.pptx]

## Slide 1
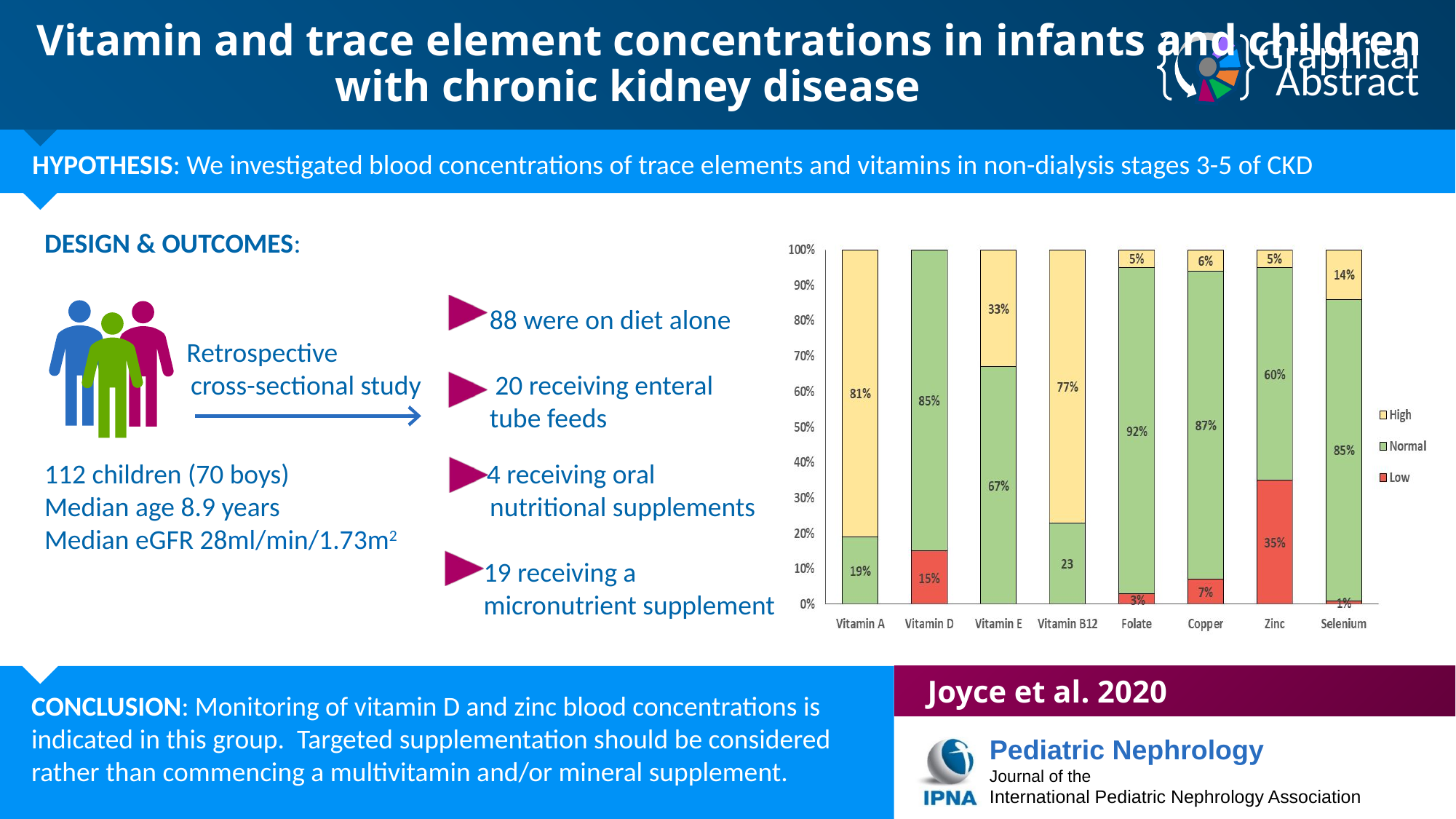

Vitamin and trace element concentrations in infants and children with chronic kidney disease
HYPOTHESIS: We investigated blood concentrations of trace elements and vitamins in non-dialysis stages 3-5 of CKD
DESIGN & OUTCOMES:
 88 were on diet alone
 Retrospective
	 cross-sectional study 20 receiving enteral
 tube feeds
112 children (70 boys) 4 receiving oral
Median age 8.9 years nutritional supplements
Median eGFR 28ml/min/1.73m2
 19 receiving a
 micronutrient supplement
Joyce et al. 2020
CONCLUSION: Monitoring of vitamin D and zinc blood concentrations is indicated in this group. Targeted supplementation should be considered rather than commencing a multivitamin and/or mineral supplement.
