# Supplementary material for: Vitamin and trace element concentrations in infants and children with chronic kidney disease
Source: Pediatr Nephrol. 2020 Apr 14;35(8):1463–70. doi: 10.1007/s00467-020-04536-0 (PMC7316696; doi:10.1007/s00467-020-04536-0)
Supplement: Supplementary file 2 — (DOCX 17 kb). [file 467_2020_4536_MOESM2_ESM.docx]

**Supplementary table** Vitamin and trace element content of micronutrient supplements
